# Molecular Mechanisms of Inhibition of Protein Amyloid Fibril Formation: Evidence and Perspectives Based on Kinetic Models

**DOI:** 10.3390/ijms232113428

**Published:** 2022-11-03

**Authors:** Igor Sedov, Diliara Khaibrakhmanova

**Affiliations:** 1 Chemical Institute, Kazan Federal University, Kremlevskaya 18, 420008 Kazan, Russia; 2 Kazan Institute of Biochemistry and Biophysics, FRC Kazan Scientific Center of RAS, 420111 Kazan, Russia; 3 Sirius University of Science and Technology, 1 Olympic Ave, 354340 Sochi, Russia

**Keywords:** amyloid fibrils, fibril formation inhibitors, protein aggregation kinetics, kinetic models, protein misfolding, protein–protein interactions, protein–ligand interactions, drug discovery, Alzheimer’s disease

## Abstract

Inhibition of fibril formation is considered a possible treatment strategy for amyloid-related diseases. Understanding the molecular nature of inhibitor action is crucial for the design of drug candidates. In the present review, we describe the common kinetic models of fibril formation and classify known inhibitors by the mechanism of their interactions with the aggregating protein and its oligomers. This mechanism determines the step or steps of the aggregation process that become inhibited and the observed changes in kinetics and equilibrium of fibril formation. The results of numerous studies indicate that possible approaches to antiamyloid inhibitor discovery include the search for the strong binders of protein monomers, cappers blocking the ends of the growing fibril, or the species absorbing on the surface of oligomers preventing nucleation. Strongly binding inhibitors stabilizing the native state can be promising for the structured proteins while designing the drug candidates targeting disordered proteins is challenging.

## 1. Introduction

Amyloid fibrils are insoluble aggregates of proteins comprising monomers non-covalently cross-linked through beta sheets. They can be formed by a huge number of proteins and peptides of different sizes, structures, and functions [1]. In humans and animals, amyloid structures are related to numerous severe disorders, including Alzheimer’s [2]. The inhibition of fibril formation with the chemical species is considered a possible amyloidosis treatment strategy. Thus, it is important to understand the mechanisms of fibrillation and its inhibition. At present, many examples of compounds showing inhibitory activity in experiments with various proteins and peptides are known, including small organic molecules [3,4,5,6], other peptides [7,8,9,10] and proteins [11,12,13], polysaccharides [14,15], synthetic polymers [16,17], carbon nanomaterials [18,19,20]. Fibril formation is a multistep process with a complicated kinetic mechanism. There are several different ways for the inhibitor molecules to retard this process. The rational design of fibrillation inhibitors requires a deep understanding of their interactions with the protein and its aggregates. Therefore, in the present paper, we review the previous studies of various inhibitors acting on different proteins in light of the molecular mechanism of action and kinetics of aggregation. A number of other reviews have also been published focusing on the various aspects of fibril formation inhibition, namely on the diversity of the chemical structure of inhibitors [3,21,22], on polymer materials showing inhibitory activity [17,23], on peptide-based inhibitors [24], or particularly on human beta-amyloid inhibitors and treatment strategies of Alzheimer’s disease [25,26,27] and transthyretin amyloidogenesis inhibitors [28].

The first part of the paper is dedicated to the kinetic description of the fibril assembly process, which is necessary to understand how the inhibitors can change the time course of fibril formation. The second part describes substances found or intentionally developed to inhibit fibril formation classified by the molecular mechanism of their interaction with protein and/or growing fibrils.

## 2. Kinetics and Thermodynamics of Amyloid Fibril Formation

### 2.1. Kinetic Models of Fibril Formation

**Figure 1:**
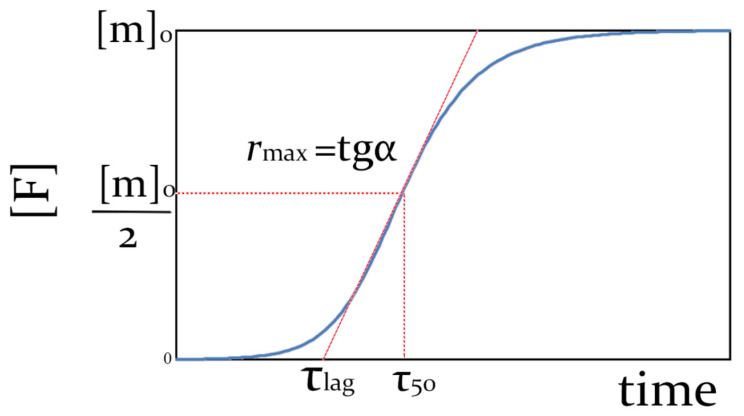
Typical time dependence of protein concentration converted into fibrils ([F]) and the quantities used to describe this curve.

The fibril formation process most commonly features a sigmoidal time course (Figure 1), which can be obtained in ThT fluorescence experiments or by any other means. It can be characterized using the lag time *τ*_lag_ defined as the onset point of the curve jump, aggregation half-time *τ*_50_ corresponding to the moment when fibril concentration reaches half of its maximum and the maximum fibril growth rate *r*_max_ at the inflection point of a sigmoid. When we speak about the inhibition of fibrillation, it means at least one of the following: an increase of *τ*_lag_, an increase of *τ*_50_, or a reduction of *r*_max_.

While some proteins readily form long and straight fibrils, others form shorter and often curved protofibrils that slowly convert into mature fibrils. Some proteins are shown to assemble into semi-flexible or “worm-like” beta-structured aggregates [29]. The same protein may form several types of fibrils under different conditions. For simplicity, we do not pay much attention to the differences between fibrils, protofibrils, and various types of “loose” or “amyloid-like” fibrils since these differences are mainly morphological, while the mechanisms of their formation and inhibition can be treated similarly.

Kinetic curves of amyloid formation can be successfully described using nucleated polymerization models viewing a fibril as a one-dimensional crystal [30]. These models suggest a mechanism involving at least primary nucleation and fibril elongation stages, with a possibility to account for secondary nucleation occurring when the surface of the already formed fibril acts as a nucleus, fibril fragmentation into smaller pieces leading to an increase in the number of fibril termini, saturated elongation and some other processes. These mechanisms lead to different dependencies of *τ*_50_ and *τ*_lag_ on the protein concentration, which can be used to distinguish between them using experimental data. The possible inhibition mechanisms are diverse; however, we can also try to deduce them based on the observed kinetic curves at different concentrations of an inhibitor and knowledge about the molecular nature of inhibitor interactions with the protein and its aggregates.

The link between the parameters of elementary steps and the observed fibrillation time course is not apparent. For example, it is incorrect to say that the increase in the lag time necessarily means inhibition of the nucleation stage [31]. Below we consider the model of nucleated fibril formation developed by Knowles et al. [32] and validated on many different systems [33,34,35,36,37]. The kinetic curves were calculated according to this model using our home-written program.

The kinetic equations for the considered model can be written in the general form:

(1) $$\begin{array}{l} \frac{d[P]}{dt}=k_{n}{\left[m\right]}^{n_{c}}+k_{2}{\left[m\right]}^{n_{2}}[F]+k_{-}[F];\\ \frac{d[F]}{dt}=2k_{+}[m][P]-2k_{off}[P]. \end{array}$$

Here [P] is the concentration of fibril particles, [F] is the total concentration of protein monomers in the fibrils and [m] is the concentration of free protein monomers. The rate constants correspond to *k_n_*, primary nucleation, *k*_+_, elongation, *k*_2_, secondary nucleation, *k*_–_, fragmentation, *k_off_*, depolymerization. Following previous works, we assume the nucleation exponents $n_{c}=n_{2}=2$. In all our calculations, a set of the rate constants taken from the literature [38] for in vitro aggregation of the Aβ42 peptide is used: *k_n_* = 10^−4^ M^−1^s^−1^, *k*_+_ = 3 × 10^6^ M^−1^s^−1^, *k*_2_ = 8 × 10^3^ M^−2^s^−1^, *k*_–_ = 3 × 10^−8^ s^−1^; *k_off_* = 2 s^−1^ (this one was chosen arbitrarily for demonstration purposes, close to the value for Aβ40 reported in [39]). Except for *k_n_* and *k*_+_, any other rate constant can be substituted by zero. The initial protein concentration is 3 μM.

**Figure 2:**
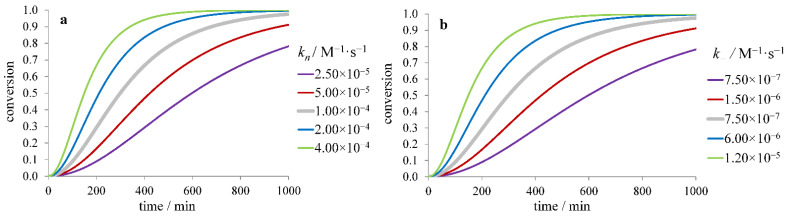
Dependence of the fibril formation time course on (**a**) nucleation (*k_n_*), (**b**) elongation (*k_+_*) rate constant for the model considering only nucleation and elongation.

Let us consider the effect of the rate constants of individual stages of fibril formation on the time dependence of fibril yield. The simplest nucleation-elongation model leads to the kinetic curves depending on the product *k_n_k*_+_ of the rate constants of nucleation *k_n_* and elongation *k*_+_. The decrease of any of these constants leads to an increase of both *τ*_lag_ and *τ*_50,_ as well as a decrease of *r*_max_ (Figure 2). The analytical solution [40], as well as numerical simulation, show that *r*_max_ is proportional to ${\left[m\right]}_{0}{}^{\frac{n_{c}}{2}+1}{\left(k_{+}k_{n}\right)}^{\frac{1}{2}}$, while *τ*_50_ and *τ*_lag_ are proportional to ${\left[m\right]}_{0}{}^{-\frac{n_{c}}{2}}{\left(k_{+}k_{n}\right)}^{-\frac{1}{2}}$.

**Figure 3:**
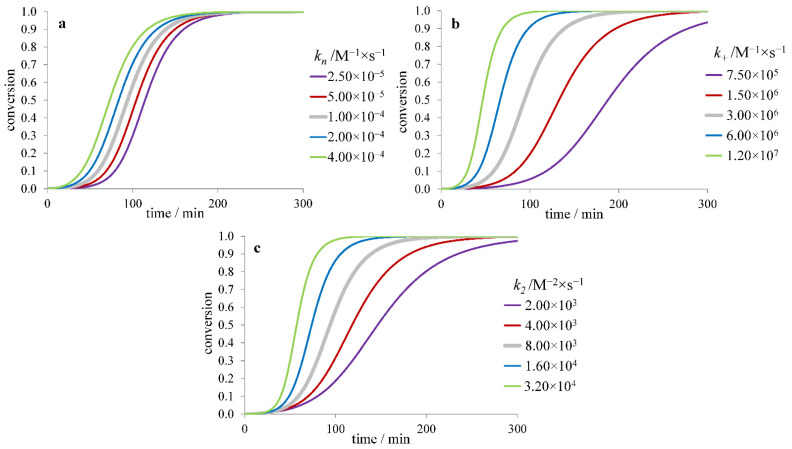
Dependence of the fibril formation time course on (**a**) nucleation (*k_n_*), (**b**) elongation (*k_+_*), and (**c**) secondary nucleation (*k_2_*) rate constant for the model with secondary nucleation.

In the model with secondary nucleation, the slowing down of primary nucleation leads to an increase of the lag time and *τ*_50_ while *r*_max_ and the difference *τ*_lag_–*τ*_50_ remain almost constant (Figure 3a). Slower elongation results in an increase of both *τ*_lag_ and *τ*_50_, as well as a decrease of *r*_max_ (Figure 3b). Slower secondary nucleation leads to a similar trend of changes but lowering its rate constant *k*_2_ leads to smaller changes than lowering the elongation rate constant *k*_+_ (Figure 3c). In addition, at *k*_2_ → 0, the kinetic curve becomes the same as for the model without secondary nucleation (Figure 2). Here, the value of *r*_max_ is proportional to ${\left[m\right]}_{0}{}^{\frac{3}{2}}{\left(k_{+}k_{2}\right)}^{\frac{1}{2}}$, *τ*_50_ and *τ*_lag_ are proportional to $k_{+}{}^{-\frac{1}{2}}$, while their dependence on [*m*]_0_, *k_n_* and *k*_2_ is more complicated (roughly proportional to ${\left[m\right]}_{0}{}^{-\frac{n_{2}+1}{2}}{\left(k_{+}k_{2}\right)}^{-\frac{1}{2}}$) [40].

**Figure 4:**
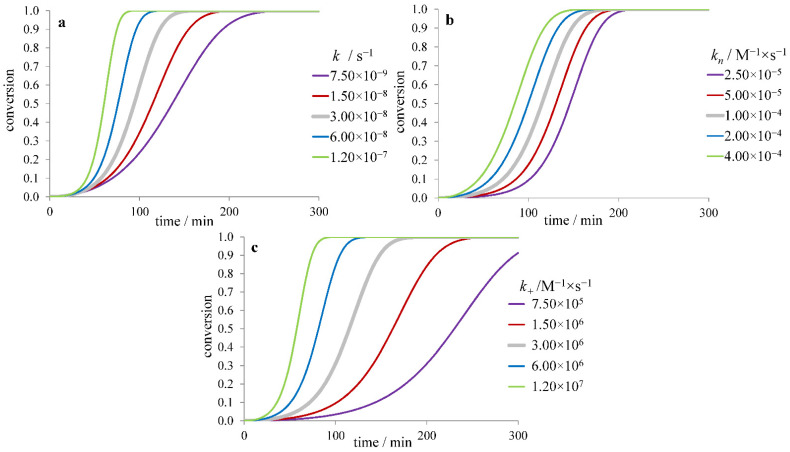
Dependence of the fibril formation time course on (**a**) fragmentation (*k_−_*), (**b**) nucleation (*k_n_*), and (**c**) elongation (*k_+_*) rate constant for the model with fragmentation and no secondary nucleation.

In the model with fibril fragmentation (without secondary nucleation) allowing spontaneous splitting of a fibril into two parts, variation of the fragmentation rate constant *k*_–_ also changes all three quantities *τ*_lag_, *τ*_50_, and *r*_max_ (Figure 4a). Fragmentation is thought to be a reason for speeding up fibril formation upon agitation of the solution [41,42]. Interestingly, this model’s variations of *k_n_* and *k*_+_ lead to changes in time courses similar to those in the model with secondary nucleation and no fragmentation (Figure 4b,c). The magnitude of *r*_max_ in this model is proportional to ${\left[m\right]}_{0}{}^{\frac{3}{2}}{\left(k_{+}k_{-}\right)}^{\frac{1}{2}}$; *τ*_50_ and *τ*_lag_ are proportional to $k_{+}{}^{-\frac{1}{2}}$ and also depend on [*m*]_0_, *k_n_* and *k*_–_ (being roughly proportional to ${\left[m\right]}_{0}{}^{-\frac{1}{2}}{\left(k_{+}k_{-}\right)}^{-\frac{1}{2}}$). At *k*_–_ → 0, the kinetic curve becomes the same as for the model without fragmentation (Figure 2). 

Other possible processes and the dependency of the rate constants on the aggregate size not considered in the described models can also affect the time course of fibrillation. For example, Kuret et al. found that end-to-end annealing, a process opposite to fragmentation, occurs upon tau protein aggregation [43]. A different kinetic behavior is observed in the systems with off-pathway aggregation leading to amorphous or globular aggregates [44,45]. Some other specific cases are considered in the following sections, including fibril formation without a lag period that cannot be described by Equation (1).

### 2.2. Fibril Formation from the Particular Form of Protein

Proteins with ordered native structures often form fibrils at denaturing conditions, suggesting that only the partially or fully unfolded form of protein monomer participates in this process [46,47]. This form (U) exists in thermodynamic equilibrium with the native monomeric protein (N). More generally, there can be several forms of protein in equilibrium, only one of which participates in fibril formation. Let the fraction of this form be denoted as α. For the simplest case, α = $\frac{\left[U\right]}{\left[U\right]+\left[N\right]}=\frac{\left[U\right]}{\left[m\right]}$. When α < 1, both the nucleation and elongation rates are smaller than in the case when any protein molecule can participate in fibrillation. Assuming a rapidly establishing equilibrium between the protein forms, we can rewrite Equation (1) as:

(2) $$\begin{array}{l} \frac{d[P]}{dt}=k_{n}{\left(\alpha [m]\right)}^{n_{c}}+k_{2}{\left(\alpha [m]\right)}^{n_{2}}[F]+k_{-}[F];\\ \frac{d[F]}{dt}=2k_{+}\alpha [m][P]-2k_{off}[P]. \end{array}$$

**Figure 5:**
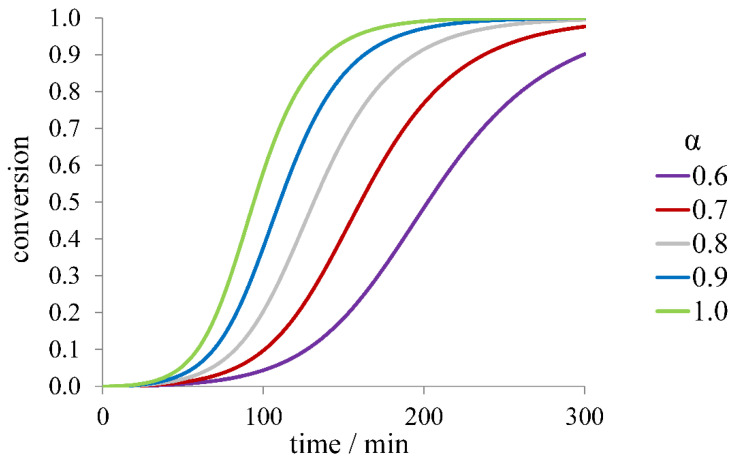
Dependence of the fibril formation time course on the fraction of aggregation-capable protein form (α) for the model with secondary nucleation and no fragmentation.

The dependence of the time course on α value at the same initial protein concentration for secondary-nucleated fibril growth (without fragmentation) is shown in Figure 5. Again, all three quantities, *τ*_lag_, *τ*_50_, and *r*_max_, change. Taking into account that introducing α into kinetic Equation (1) is equivalent to multiplying the rate constants by α in the respective powers, we deduce that *r*_max_ scales as $\alpha^{\frac{n_{2}+1}{2}}$ ($\alpha^{\frac{3}{2}}$ if *n*_2_ = 2), while *τ*_50_ and *τ*_lag_ scale approximately as $\alpha^{-\frac{n_{2}+1}{2}}$.

### 2.3. Thermodynamic Equilibrium of Fibril Formation

The process of amyloid fibril formation is reversible, although the equilibrium monomer concentration is small for many proteins [48]. This concentration equals the thermodynamic equilibrium constant of fibril dissociation *K_F_* if only one form of the monomer is present and participates in aggregation. Thus, the degree of conversion of protein into fibrils after prolonged incubation is not strictly equal to unity and is governed by this constant. The simplest way to account for the reversibility of aggregation in a kinetic model is to introduce the depolymerization rate constant $k_{off}$ of the detachment of protein monomers from the fibril termini. From Equation (1), it follows that at an infinite time when fibrils concentration becomes constant, the equilibrium monomer concentration equals the ratio of depolymerization and fibril elongation rate constants:

(3) $${\left[m\right]}_{eq}=\frac{k_{off}}{k_{+}}=K_{F}$$

**Figure 6:**
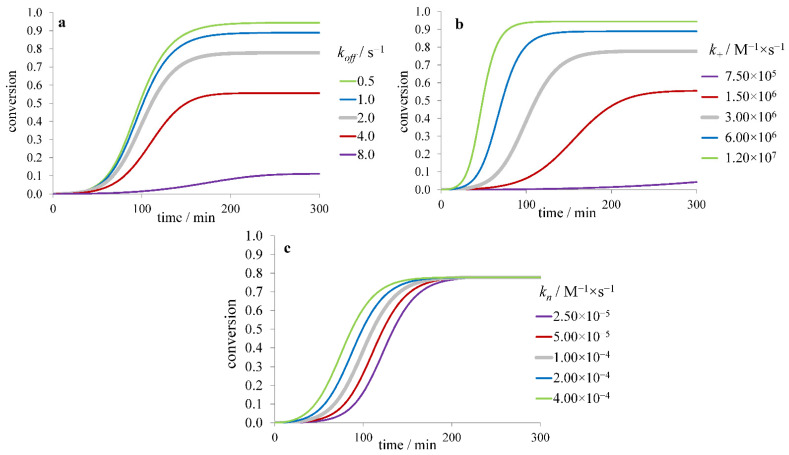
Dependence of the time course on (**a**) depolymerization (*k_off_*), (**b**) nucleation (*k_n_*), (**c**) elongation (*k_+_*), rate constant for the model of reversible fibril formation with secondary nucleation.

Figure 6a shows the dependence of fibril formation kinetics on the value of $k_{off}$ for the model with secondary nucleation. With increasing depolymerization rate, the equilibrium conversion degree decreases and *τ*_50_ increases. The decrease of elongation rate constant in the case of significantly reversible fibrillation also lowers the fibril yield but leads to larger changes of *τ*_lag_ and *τ*_50_ (Figure 6b). In contrast, the change in the nucleation rate constant does not affect the fibril yield (Figure 6c).

If the protein exists as an equilibrium mixture of two conformers, e.g., the folded and unfolded states, and only one conformer with its equilibrium molar fraction α participates in reversible fibril formation, then the fibril yield becomes lower with decreasing α even at the constant values of *k*_+_ and *k_off_*. In such a case, the total (folded and unfolded) monomer concentration at equilibrium is given by:

(4) $${\left[m\right]}_{eq}=\frac{K_{F}}{\alpha }=\frac{k_{off}}{\alpha k_{+}}$$

**Figure 7:**
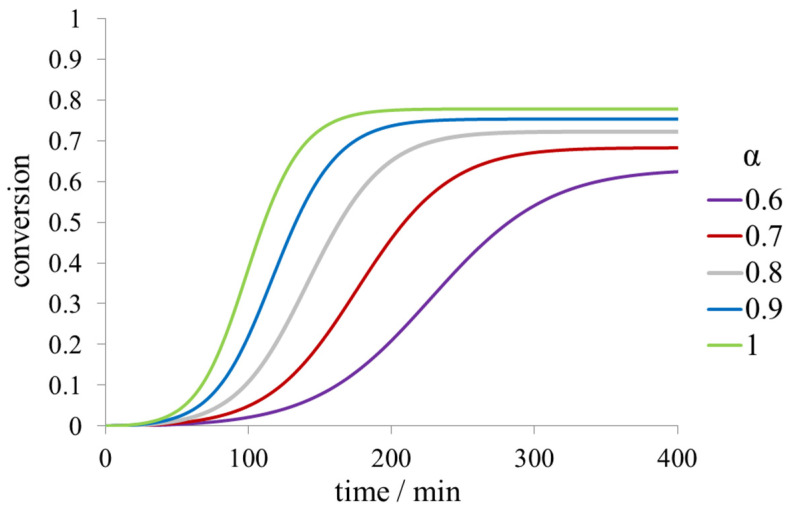
Dependence of the time course on the fraction of aggregation-capable protein form (α) for the model of reversible fibril formation with secondary nucleation and depolymerization.

The kinetic curves for different values of α are given in Figure 7. The scaling exponents of *τ*_lag_, *τ*_50_, and *r*_max_ with respect to α depend on the value of $k_{off}$.

### 2.4. Fibril Formation without a Lag Period

There are also numerous examples of systems in which fibril formation occurs without a lag period. Such behavior is typical for certain proteins like transthyretin [49,50], human [51] and bovine [52] serum albumin, chicken egg-white ovalbumin [38], α_s2_- and κ-casein [53,54], acylphosphatase [55], mouse prion protein [56,57], bacterial lipase [58], disulfide cross-linked dimers of human beta-amyloid [59,60], antimicrobial peptide uperin from frog skin [61] or 22-residue K3 peptide of β_2_-microglobulin [62]. Other proteins can show no lag when forming fibrils under specific conditions, e.g., human beta-amyloid in the presence of sodium dodecylsulfate [63] or a small concentration of hexafluoroisopropanol [64], β_2_-microglobulin at certain pH value [65,66]. It should also be noted that fibrillation without a lag sometimes leads to fibrils or protofibrils with a morphology different from classical long and straight aggregates, e.g., worm-like assemblies of β_2_-microglobulin [66] or mouse prion [57].

One possibility to explain such kinetics is the very rapid formation of fibril nuclei. This can be seen by comparing the curves corresponding to the different *k_n_* values in Figure 2. Large values of the primary nucleation rate constant lead to the essentially unnoticeable lag. However, for many systems that show lag-free fibrillation, there is strong evidence that fibril formation proceeds without nuclei formation: no lag is observed even at very low protein concentrations, and the process does not accelerate in the experiments involving seeding with preformed fibrils. This appears to be the case for albumins in physiological conditions [51,52]. It was also hypothesized that the surface of the sample container can be saturated with fibril seeds on which fibrils subsequently grow [38]. In such a case, the initial rate of fibril formation is given by:

(5) $$v_{0}=2k_{+}[P][m{]}_{0}$$

i.e., scales linearly with monomer concentration, which was observed in many systems. Another possibility is a slow rate-limiting conformational change of a protein succeeded by fast aggregation, which also leads to first-order kinetics. Alternatively, a different kinetic mechanism of fibril growth could be responsible for such behavior [67]. The crystallization-like model (CLM) suggested by Martins et al. [67] is able to describe both sigmoidal and lag-free hyperbolic aggregation kinetics with a single equation (given using our notation):

(6) $$\begin{array}{c} \frac{\left[F\right]}{{\left[F\right]}_{\max}}=1-\frac{1}{k_{b}\left(e^{k_{g}\sigma_{0}t}-1\right)+1};\\ \sigma_{0}=\frac{{\left[m\right]}_{0}-K_{F}}{K_{F}}. \end{array}$$

Here, σ_0_ is the supersaturation of protein solution relative to the equilibrium with fibrils,

(7) $${\left[F\right]}_{\max}={\left[m\right]}_{0}-K_{F},$$

which is the maximum possible (equilibrium) concentration of protein in the fibrils, and *k_g_* and *k_b_* are the growth and nucleation-to-growth rate constants, respectively. The main difference between the fibril formation mechanism serving as the basis of this model from the models considered above is the adsorption of protein monomers by the whole surface of the fibrils and their fast migration to the fibril ends instead of a direct interaction of the fibril ends with monomers. Thus, the rate of fibril growth becomes proportional to the total concentration of protein monomers in the fibrils ([F]) and not the concentration of fibril particles ([P]). The resulting differential equation can be integrated analytically, which leads to Equation (2). The time dependence of fibril concentration is sigmoidal at *k_b_* > 0.5 and hyperbolic otherwise. In hyperbolic mode, the maximum aggregation rate is observed at *t* = 0 and equals

(8) $$r_{\max}=v_{0}={\left[F\right]}_{\max}k_{b}k_{g}\sigma_{0}=\frac{k_{b}k_{g}}{K_{F}}{\left({\left[m\right]}_{0}-K_{F}\right)}^{2}$$

At low $K_{F}$ values, this rate scales as the square of initial protein concentration and is proportional to both fibril growth and nucleation-to-growth rate constants. The value of *τ*_50_ is given by

(9) $$\tau_{50}=\frac{1}{k_{g}\sigma_{0}}\ln\left(\frac{1}{k_{b}}+1\right)$$

and should be approximately reciprocal with respect to the initial protein concentration.

The hyperbolic aggregation curves are also observed in seeded experiments in which a certain concentration of the preformed fibrils is added to the protein solution at the initial moment of time. The curve becomes hyperbolic only at high seed concentrations when the nucleation processes do not change the number of fibril particles significantly. The initial rate of fibril growth here is also given by

(10) $$v_{0}=2k_{+}[P][m{]}_{0}$$

Such a simple dependence provides a convenient way for experimental characterization of the effect of various inhibitors or other factors on fibrillation kinetics.

## 3. Effect of Inhibitor Binding on Amyloid Fibril Formation

The models considered above greatly help to understand the influence of various compounds on the fibril formation kinetics. It should be understood, however, that the addition of an inhibitor is not just an alteration of the rate constant values. Most of them bind non-covalently to a protein or its aggregates, thus changing the kinetic scheme of fibril formation. Below, we consider different possible molecular mechanisms of inhibitor action with experimentally confirmed examples.

### 3.1. Inhibitors Influencing the Protein Monomer

This is likely the broadest group of systems. The simplest option is a 1:1 binding of a monomeric protein with an inhibitor I leading to the formation of a complex unable to participate in both nucleation and fibril growth processes. Let us assume that the free inhibitor and protein quickly reach equilibrium with the complex, and this equilibrium cannot be shifted by the fibril formation process. This is reasonable since fibril formation takes tens of minutes, even for the fastest aggregating proteins, and, in some cases, may take many days. Then the fraction of unbound monomeric protein

(11) $$\alpha =\frac{1}{1+K_{a}\left[I\right]}$$

where *K_a_* is the binding constant, and [I] is the equilibrium inhibitor concentration. The kinetics of fibril formation can be described by Equation (2), where the protein monomer concentration [*m*] from Equation (1) is replaced with α[*m*]. In general, α can change during the course of aggregation with changing monomer concentration, but at sufficiently large concentrations of inhibitor, only a small part of its molecules bind to the protein, so [I] ≈ [I]_0_ and α can be assumed to be constant ([I]_0_ is the concentration of inhibitor initially added to the system). Then, the dependence of kinetic curves for the secondary-nucleated process without fragmentation and depolymerization on α is like what is shown in Figure 5, and the scaling laws are as discussed above (*r*_max_ scales as $\alpha^{\frac{n_{2}+1}{2}}$, *τ*_50_ and *τ*_lag_ scale approximately as $\alpha^{-\frac{n_{2}+1}{2}}$). In seeded experiments, the initial rate should be proportional to α. The inhibitor-bound protein may also participate in fibril growth with different (likely lower) rate constants ($k_{+}’$). Then, the growth rate in Equation (1) can be written as $2(k_{+}\alpha +k_{+}’(1-\alpha ))[m][P]$, which can also be regarded as the modification of the rate constant value. The same is true for the nucleation involving complexes, but the expressions relating *k_n_* and *k*_2_ to α will be more complicated, as well as the scaling laws for the kinetic curve parameters. Moreover, the magnitude α can be introduced when the protein binds any number of inhibitor molecules with any values of binding constants.

As mentioned above, natively folded proteins usually form fibrils from their partially or completely unfolded form, which is in equilibrium with the native form. The native or, less commonly, the unfolded form can bind the inhibitor. In this case, we can also use Equation (2), with α being the fraction of the free unfolded form of monomeric protein, which can be expressed through the inhibitor concentration and the equilibrium constants of binding and unfolding.

Change in the value of α always affects the thermodynamics of equilibrium fibril formation, decreasing the fibril yield as shown in Figure 7; nevertheless, this can leave unnoticed in experiments in the case of weak binding or low *K_F_* values. Hence, monomer-binding species act as thermodynamic inhibitors of fibrillation [68], but of course, they also affect the kinetic parameters of this process. Strong binders changing the fibril yield should be able to disrupt the preformed fibrils at least partially. In reality, this can be a very slow process due to their huge kinetic stability (very low *k_off_* values) [69].

#### 3.1.1. The Effect of Inhibitor Binding to the Protein Monomer

Fibrillation of a number of natively disordered proteins is linked to currently incurable diseases, including human beta-amyloid peptides Aβ42 and Aβ40 involved in Alzheimer’s disease. Hence, the development of efficient inhibitors of the fibrillation of these proteins attracts the biggest attention. While the native form of folded proteins can be stabilized by tight binding with both other proteins and small organic molecules, disordered proteins usually show no high affinity to the small molecules. There are no approved small-molecule drugs targeting such proteins.

One well-characterized example of Aβ42 inhibitors [70] is biphenyl-2-yl-(7-nitro-benzo [1,2,5]oxadiazol-4-yl)-amine (10074-G5) with a 6 μM affinity to the monomer determined using biolayer interferometry, which is considerably weaker affinity than of many drugs targeting structured proteins. Nevertheless, it was shown to influence the kinetic parameters of the microscopic steps of fibril formation and decrease the fibril yield in agreement with the kinetic model discussed above. Lysine-specific molecular tweezers [71,72] were designed and shown to inhibit fibrillation of different disordered proteins, including beta-amyloid, tau protein, α-synuclein, islet amyloid polypeptide (IAPP), as well as structured proteins, such as β2 microglobulin, insulin, and transthyretin. Disaggregation of the preformed fibrils was also reported. These anionic molecules bind the lysine residues in protein monomers, impeding the processes of fibril nucleation and growth. Their binding to the lysines in the fibrils likely leads to the destabilization of favorable contacts with other residues.

**Figure 8:**
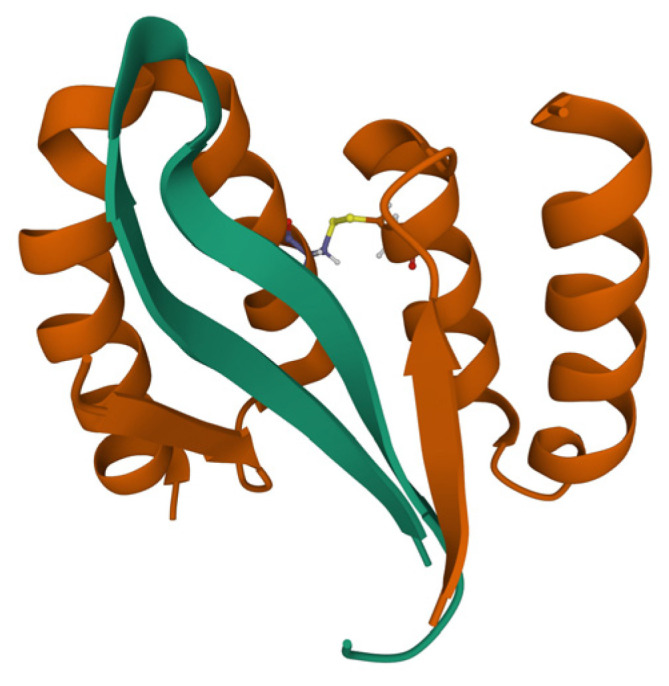
Complex of disulfide-linked dimeric affibody protein ZAβ3 (brown) with Aβ40 in β-hairpin conformation (green). PDB ID: 2OTK.

The affinity of disordered proteins to other proteins can be much higher. Stahl et al. engineered a disulfide-linked dimeric affibody protein Z_Aβ3_ [73] with a 17 nM affinity to Aβ40. It stabilizes a beta-amyloid in β-hairpin conformation, blocking it from two sides, and preventing further β-sheet extension (Figure 8). The affibody was shown to inhibit Aβ40 fibrillation, with almost no fibrils forming at a 1:1 inhibitor/beta-amyloid molar ratio. Later engineering attempts led to an even more potent binder, ZSYM73, with a 300 pM affinity [74].

There are also reports of a negatively charged ulvan polysaccharide [14,75] and positively charged poly- and oligosaccharides derived from chitosan [76,77,78], thus inhibiting fibril formation of Aβ40, Aβ42, or α-synuclein, and in some cases, disaggregating preformed mature fibrils. It is possible that this effect is caused by the interactions with the Aβ monomer. At the same time, other polysaccharides, especially sulfated glycosaminoglycans, promote the fibrillation of beta-amyloid [17,76]. This was attributed to the different spatial charge distribution making some polysaccharides able to serve as scaffolds facilitating the fibril assembly, while others just sequester protein monomers [76]. Synthetic polymers can also show different effects on fibril formation. Inhibitory activity against the aggregation of disordered proteins, such as beta-amyloid, α-synuclein, and IAPP, was observed for some anionic and cationic aromatic polymers [16], including PAMAM [79,80,81] and poly(propylene imine)-maltose [82] dendrimers, polymer-coated inorganic nanoparticles [83,84] and other polymeric molecules and materials [17]. In most studies, their activity is explained by sequestering protein monomers; fibril disaggregation ability was also reported for some polymers. The inhibitory and disaggregating effect of graphene oxide, graphene, carbon nanotubes, and fullerene derivatives on the fibrillation of beta-amyloid [18,19,20,85,86,87], IAPP [88], α-synuclein [89,90] is linked to the binding of protein monomers, despite carbon nanoparticles also binding to oligomers that hamper their elongation and secondary nucleation [89].

**Figure 9:**
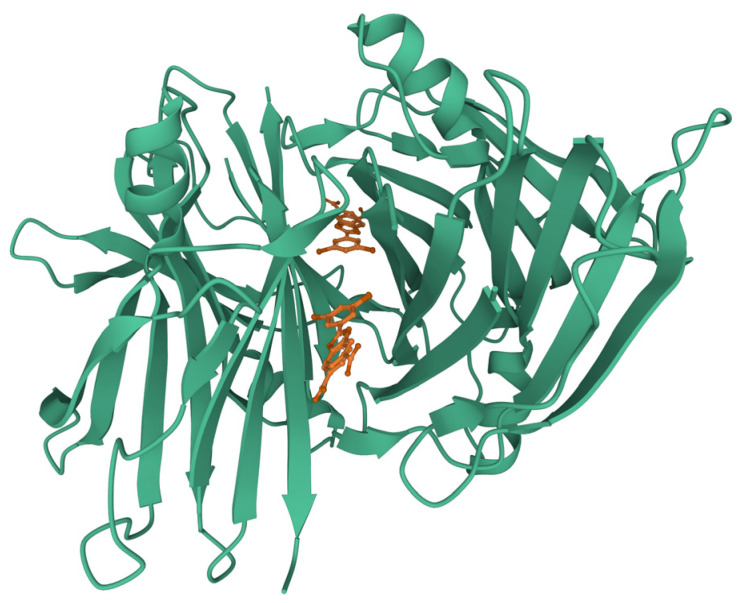
Complex of tafamidis with transthyretin tetramer. PDB ID: 3TCT.

For the structured proteins, fibril formation can be inhibited by almost any ligand or other protein binding to their native state, which leads to a decrease in the equilibrium amount of the unfolded state prone to aggregation. For proteins composed of several chains, ligands may stabilize the quaternary structure and prevent the dissociation of monomeric chains that can convert into fibrils. Numerous studies report examples of the native state-stabilizing inhibitors for different proteins, including lysozyme [91,92,93], acylphosphatase [94], prion protein [95,96], immunoglobulin light chain variable domain [97], insulin [98,99], albumins [100,101], and others. Good prospects of the native state stabilizers as drug candidates can be shown in the example of transthyretin, a natively tetrameric protein that can dissociate into monomers. The latter was shown to form fibrils [102] engaged in the pathogenesis of amyloid polyneuropathy and cardiomyopathy. The inhibition of its aggregation by various nonsteroidal anti-inflammatory drugs [103], phenols and polyphenols [104,105,106,107] and a number of other compounds [108,109,110,111] is caused by their binding to the tetrameric state. The studies of transthyretin binders culminated in the discovery of the clinically approved drug, tafamidis [112], a first-in-class medication with 2 nM affinity to the tetramer (see Figure 9 for the structure of complex).

An interesting example of inhibitors binding the unfolded state of structured proteins, such as bovine serum albumin [113] or lysozyme [114,115], and preventing its further aggregation is graphene oxide and graphene nanoparticles.

#### 3.1.2. The Effect of Protein Stabilizers and Denaturants

A special case is the shift of equilibrium between the native and unfolded form of protein under the action of non-binding substances, which can be protein denaturants (numerous organic solvents [116,117]), stabilizers (sugars, polyols, some inorganic salts [118,119]), crowding agents, or micelle-forming detergents. In the case of fibril formation from the unfolded form of the natively structured protein, the addition of denaturants leads to the increase of α value, which is a technique sometimes used to accelerate this process. Since such compounds are added at rather large concentrations, they may also change the rate constants of any step of fibrillation. In addition, some of them can change the values of pH and ionic strength, which also influence the rate and equilibrium constants. For some proteins, conformation equilibria between different fully or partially unfolded forms become shifted, some of which cannot be prone to aggregation or produce non-fibrillar aggregates. As a result, it is difficult to predict whether some well-known denaturant promotes or prevents the fibril formation of a particular protein without the experimental study.

Vice versa, stabilizers decrease the fraction of the unfolded forms. In most cases, they inhibit the fibril formation of globular proteins with a well-defined definite native structure. Mono- and disaccharides, such as sucrose, trehalose, or glucose and polyhydric alcohols, inhibit aggregation of hen egg-white lysozyme [120,121], insulin [121,122], α-lactalbumin [123], bovine serum albumin [124], Vλ6 protein [125] by stabilizing their native state. Another well-known osmolyte, trimethylamine N-oxide (TMAO), inhibits insulin [126,127] fibril formation. The effect of osmolytes on the fibrillation of intrinsically disordered proteins is often the opposite. Various sugars are shown to induce β-amyloid fibrillation [128,129] while fructose does not [129], and trehalose inhibits it and even dissolves preformed Aβ40 amyloid aggregates [130]. In the presence of moderate concentrations of TMAO, α-synuclein greatly enhances the rate of formation and yield of fibrils, while at high concentrations, fibrillation becomes suppressed in favor of the formation of globular oligomers [131]. TMAO also accelerates the aggregation of beta-amyloid [132] and tau protein [133]. Disordered proteins exist as ensembles of different conformations with different populations. These populations change upon the addition of osmolytes which generally favor more compact and structured states that can be more prone to nucleation and attaching to fibril ends [121], but there can be some exclusions.

Consequently, denaturants are more likely to inhibit than accelerate fibril formation from disordered proteins. Aβ42 fibrillation is inhibited by urea [134,135] and guanidinium chloride at concentrations above 1 M [135,136] due to a decrease in the rate of both primary and secondary nucleation. Interestingly, at low concentrations, guanidinium chloride act as an osmolyte, increasing the rate of beta-amyloid fibrillation, likely due to the electrostatic interactions with the ions formed upon its dissociation. α-synuclein fibrils undergo full depolymerization to monomers after prolonged incubation in a solution containing more than 3.5 M of urea [137]. Urea also suppresses tau protein fibrillation [138]. In the presence of fluorinated alkanols, such as 2,2,2-trifluoroethanol (TFE) and 1,1,1,3,3,3-hexafluoro-2-propanol (HFIP), a bell-shaped dependence of the rate of fibril formation on denaturant concentration is observed for Aβ40, Aβ42 [136,139,140], and α-synuclein [140,141]. At moderate concentrations of these alcohols, partially folded intermediates are formed, which form fibrils quicker than the disordered states. At such conditions, fibril formation becomes more favorable thermodynamically. Beta-amyloid was shown to aggregate even at nanomolar concentrations, which is below the equilibrium monomer concentration for its fibrils in the absence of additives (*K_F_*) [139]. High concentrations of fluorinated alcohols stabilize α-helical conformation making fibril formation unfavorable both thermodynamically and kinetically. Methanol, ethanol and propanol can also stabilize a partially folded state and decrease the fibrillation lag time of α-synuclein at moderate concentrations, while at higher concentrations, they promote the formation of non-fibrillar oligomers [140]. Pure TFE and HFIP are known to disaggregate fibrils of beta-amyloid and other proteins [142,143,144].

Natively folded proteins can accelerate fibril formation under the action of denaturants at moderate concentrations. Lysozyme fibrils can be obtained in the presence of 2–4 M guanidinium chloride within several hours instead of several days at 50 °C [145] due to the increase in the fraction of the unfolded form. At the concentration of 5 M and higher, lysozyme becomes completely unfolded. The unfolded state becomes so thermodynamically favorable that concentrated guanidinium chloride prevents the protein from fibrillation and is able to dissolve preformed fibrils. Fibrils of *α*-lactalbumin [137] and many other proteins [146] can also be depolymerized with guanidinium salts. For the fibril formation of β-lactoglobulin increase in the presence of urea, the maximum yield and the minimum lag time were observed at 5 M urea concentration [147]. Denaturants methanol, ethanol, and DMSO [148] were also shown to promote the fibrillation of β-lactoglobulin. Moderate concentrations of TFE were shown to induce fibrillation of acylphosphatase [149], human stefin B [150], and insulin [151]; at high concentrations, no fibrils are formed.

On the other hand, FF domain of the URN1 splicing factor, a highly α-helical protein, loses the ability to form fibrils even at moderate concentrations of TFE, which stabilizes its native helical structure [144]. The lag time of bovine insulin aggregation at pH 4.0 in the presence of methanol, ethanol, or isopropanol increases with the alcohol concentration up to 30–40 volume % and starts to decrease at higher concentrations [152]. The fibril yield greatly and steadily decreases with increasing alcohol concentration due to the parallel formation of non-fibrillar aggregates, which was confirmed by AFM imaging. In contrast, the study of aggregation of insulin in 0.1 M NaCl at pH 1.9 [153] showed the lowest lag time at 10 weight % of ethanol which increased at its higher concentrations. The addition of increasing amounts of guanidinium chloride to insulin first increases and then decreases the overall rate of fibril formation, as characterized by the lag time [154] and particularly the rate of elongation [126]. These results are related to the complex equilibria between insulin hexamer, tetramer, dimer, monomer, and the unfolded forms of monomer shifted by the organic cosolvent as well as pH value [155,156].

#### 3.1.3. The Effect of Crowding Agents and Detergents

Crowding agents such as polysaccharides (dextran, mannan, glycogen, Ficoll), polyethylene glycols, or high concentrations of other proteins accelerate fibrillation of both structured and disordered proteins: β-lactoglobulin [157], lysozyme [158], β-amyloid [158,159], α-synuclein [160,161], tau protein [162], human prion protein [162], and many others. Unlike charged polymers, they cannot tightly bind protein monomers or aggregates. Their influence is usually attributed to the acceleration of fibril nucleation and growth by the excluded volume effects [163]. However, oligomeric proteins might be stabilized in their native state by crowding agents and have a reduced aggregation rate [164].

Small-molecule detergents may have opposing effects on fibril formation. Sodium dodecylsulfate (SDS) micelles prevent fibril formation of Aβ42 and Aβ40 and convert them into the α-helical structures in a micellar environment [63,165,166], but at lower concentrations of SDS, the acceleration of aggregation is possible [167,168]. A similar difference in the influence of submicellar and micellar concentrations of SDS was also observed for concanavalin A fibrillation [169] and SCR3(18-34) peptide [170]. Low concentrations of SDS and sarkosyl detergent induced fibril formation from a prion protein by the destabilization of its native state [171,172]. Short-chain phospholipids in submicellar concentrations accelerated the fibrillation of apolipoprotein C-II by inducing its conversion into a tetramer serving as a nucleus for further fibril growth [173]. At the same time, micelles of the same phospholipid suppressed aggregation due to their favorable interactions with the protein.

### 3.2. Inhibition of Fibril Growth by Binding to Aggregate Ends

Inhibitor molecules can bind to the ends of fibrils impeding their ability to further growth. When the inhibitor interacts only with the terminal protein units, we can expect that the binding constant $K_{b}$ will not depend on the aggregate size. For equilibrium binding, the fraction of the termini free from inhibitor molecules is thus given by”

(12) $$\beta =\frac{1}{1+K_{b}\left[I\right]}$$

Since fibrils are quite long and the concentration of fibril particles binding the inhibitor ([P] in Equation (1)) is small, it can often be assumed that $\left[I\right]\approx {\left[I\right]}_{0}$, then the β value does not significantly change during the whole process. In the secondary nucleation model without fragmentation and depolymerization, this changes only one term in Equation (1), which becomes $2k_{+}\beta [m][P]$ instead of $2k_{+}[m][P]$. Thus, such binding is equivalent to a decreasing *k*_+_ (see Figure 3b), *r*_max_ scales as $\beta^{\frac{1}{2}}$, *τ*_50_ and *τ*_lag_ scale as $\beta^{-\frac{1}{2}}$. In seeded experiments, the initial rate should be proportional to β. We do not explicitly consider binding to both protein monomers and aggregates here, but it should be understood that this is possible. Inhibitors may also bind to aggregates with a size-dependent affinity and may just decrease the rate of monomer addition to the end of fibril but not completely suppress it. All of these options complicate the kinetic models.

One possible strategy for the design of fibril growth inhibitors is to develop peptides containing specific amino acid sequences, making them capable of selective binding with the ends of fibrils and a disruptive element blocking, thus furthering aggregation [174]. Self-recognition of the proteins or peptides during fibril assembly allows for the use of specific parts of their amino acid sequences as the binding element. For beta-amyloid, amyloidogenic fragment 17–21 KLVFF is itself able to bind to fibril ends with a micromolar affinity and significantly reduce the fibril formation rate [7]. Its conjugates with polyethylene glycol or dendrimers containing several copies of the peptide are much more potent inhibitors, with up to 100 pM affinity to the fibrils [175,176]. Peptides containing alpha,alpha-disubstituted amino acids [177,178], D-aminoacids instead of L-enantiomers [9,179,180,181], substituted proline residues [182], N-methyl amino acids in alternating positions [183], retro-inverso peptides with flipped CO and NH groups [184,185], many different peptidomimetics [186], and glycopeptides [187] were synthesized and shown to inhibit Aβ42 and Aβ40 fibrillation. Modification of the whole aggregating protein is also used. In order to inhibit α-synuclein aggregation, a chimeric inhibitor comprising a globular protein with 41.5 kDa molecular weight fused to the C-terminus of the α-synuclein monomer has been developed [188]. α-synuclein, with a preformed hairpin between cysteine residues, was also shown to inhibit the wild-type synuclein fibril elongation [189]. When the modified and wild-type proteins were fused, a much more potent inhibitor was obtained. A completely different aggregation-prone protein can also act as an inhibitor; for example, the mouse prion protein [190] or the molecular chaperone clusterin [191] binds to the ends of growing fibrils of beta-amyloid, inhibiting their elongation.

The advantage of inhibitors binding to fibril ends, which are present at very low concentrations, is that they can be active even at a small molar ratio to the protein. In contrast, in order to suppress aggregation using the inhibitors binding to protein monomer or fibril surface—they should be taken in at least a 1:1 molar ratio to protein, even in the case of strong binding. For example, the fused synuclein-protein inhibitor [188] had one μM affinity to synuclein fibrils according to the inhibition data. At this concentration, it reduced the rate of fibril formation of 50 μM synuclein seeded with one μM preformed sonicated fibrils approximately twice. The value of IC_50_ in seeded elongation experiments for another above-discussed fused inhibitor obtained from wild-type and modified synuclein was 11 nM [189], and it was active against the fibrillation of protein with a thousands-times higher concentration.

Binding to the fibril ends shifts the equilibrium of the protein aggregate mixture to the side of lower-sized aggregates. At low inhibitor concentrations, this has no effect on the total yield of aggregates. However, at high concentrations, strong binders may break the fibril down to the smallest possible fragments or even to monomers if they are able to bind them. There is experimental evidence that some peptide-based inhibitors at high concentrations are able to disrupt preformed fibrils [192,193]. Hypothetically, molecules binding only to aggregates, but not to the protein monomers, may increase the number of aggregates if the protein has a small degree of equilibrium fibril conversion (large *K_F_* value). In this unusual case, such a molecule will act as a thermodynamic promoter of fibril formation.

Some small non-peptide molecules can selectively bind to aggregate ends. Since amyloidogenic fragments of proteins are often rich in aromatic residues, a hypothesis about the governing role of π-stacking interactions in amyloid formation was suggested [4] and led to numerous studies of various aromatic compounds as possible inhibitors. The most attention was paid to polyphenolic compounds [194] like resveratrol [195,196,197], epigallocatechin gallate [196,198,199], curcumin [200,201], rosmarinic acid [202,203], quercetin [204,205,206], tetracycline derivatives [207], and other species [208,209,210,211] that show significant inhibiting activity with respect to beta-amyloid fibrillation in vitro. Binding to aggregates can be proven by the presence of inhibiting activity at a low inhibitor/protein molar ratio [212]. Phenol itself does not inhibit the formation of fibrils; all potent inhibitors comprise at least two benzene rings with at least three hydroxyl groups attached to them, which allows for the adoption of the conformations necessary for non-covalent interaction with the β-layers of amyloids. 

In a recent cryo-EM study [198], epigallocatechin gallate molecules were found to stack on the surface of tau protein amyloid fibrils disrupting the fibril architecture by curving the fibril stack. This can lead to a decrease in the rate constant of fibril elongation *k*_+_ and an increase in the depolymerization rate constant $k_{off}$. Unfortunately, polyphenols are characterized by low bioavailability and high reactivity—many of them are so-called pan-assay interference compounds (PAINs) that interact with numerous biological targets and appear to be inactive in clinical studies.

Inorganic ions may also follow this mechanism of inhibition. It was hypothesized that the Cu^2+^ ion inhibiting fibril elongation blocks the ends of growing beta-amyloid fibrils [213]. However, it may also interact with monomeric proteins, as was shown for the Zn^2+^ ion [214].

### 3.3. Inhibition of Secondary Nucleation by Binding to the Fibril Surface

Inhibitors can bind to the aggregates so that a part of their surface becomes inaccessible for protein monomers and cannot serve as a center of secondary nucleation. The equilibrium binding is expected in most cases; however, there is evidence of the non-equilibrium inhibition of Aβ42 aggregation by a molecular chaperone, which slowly binds to the fibril surface [215]. The rate of secondary nucleation will then be proportional to the fraction *γ* of the fibril surface accessible for nucleation. We can relate *γ* to the inhibitor and fibril concentrations using some adsorption models. For example, for Langmuir adsorption with the assumption that absorbed inhibitor molecules can block all possible nucleation sites,

(13) $$\gamma =1-\frac{K_{L}[I]}{1+K_{L}[I]}=\frac{1}{1+K_{L}[I]},$$

where *K_L_* is the Langmuir constant. In the above-considered model (Equation (1)), this will change only one term, which becomes equal to $k_{2}\gamma {\left[m\right]}^{n_{2}}[F]$. The concentration of fibrils and their surface area undergo significant change during their growth, and during the lag period, they are very small. Thus, the significant inhibition of secondary nucleation can be achieved by strongly binding inhibitors, even at substoichiometric concentrations. In such a case, the *γ* value quickly grows up when the lag time is over due to the depletion of the unbound inhibitor form. On the other hand, at sufficiently large concentrations of inhibitor, we can assume [I] ≈ [I]_0_, as *γ* does not change during the aggregation and inhibition of secondary nucleation becomes equivalent to scaling *k*_2_ (see Figure 3c). Then, in the absence of fragmentation and depolymerization, *r*_max_ scales as $\gamma^{\frac{1}{2}}$, *τ*_50_ and *τ*_lag_ are roughly proportional to $\gamma^{-\frac{1}{2}}$, but at very low *γ* values when *k*_2_ → 0, and these quantities approach their values for the model without secondary nucleation. In the seeded experiments, the initial rate should not depend on *γ*.

A number of examples of secondary nucleation inhibition have been reported. The molecular chaperone Brichos domain from prosurfactant protein C changed the time course of human beta-amyloid Aβ42 aggregation in a way that corresponds to a decrease of *k*_2_ [12]. It was proven to bind to the surface of fibrils using TEM and had a 40 nM binding constant according to the SPR measurements. Triggering receptor expressed on myeloid cells 2 (TREM 2) slowed the Aβ-peptides fibril formation but was shown not to bind to the protein monomers [36]. The effect was attributed to the inhibition of secondary nucleation. Globular aggregates of covalently linked Aβ40 dimer were found to inhibit secondary nucleation of wild-type Aβ40 by binding to the surface of its fibrils with 160 nM affinity [45]. A monoclonal antibody aducanumab targeting soluble Aβ oligomers and binding the N-terminus of peptides shows inhibition of secondary nucleation, while some other Aβ-binding antibodies do not [216]. Co-chaperonin prefoldin was shown to interact with both surface and ends of IAPP fibrils [217] based on both kinetic, NMR and cryo-EM data. Thus, it also affects the elongation rate. Moreover, some species seem to inhibit both primary and secondary nucleation. Knowles et al. [218] showed the ability of transthyretin to simultaneously decrease the rates of primary and secondary nucleation of the αβ-peptide. Primary nucleation was hypothesized to be impeded by the binding of the inhibitor to the oligomers before their conversion to the shortest fibrils able to elongate. The same group has also found a number of small molecules inhibiting both primary and secondary nucleation of human beta-amyloid fibrils [6] and secondary nucleation of α-synuclein oligomers [219]. Binding at the fibril surface is expected for molecular probes of fibril formation, such as Thioflavin T and other fluorescent dyes [220]. Monitoring the fluorescence signal of Thioflavin T is the most common method to obtain the kinetic curves of fibrillation. Regardless of a quite strong binding to the fibrils with less than a 1 μM affinity for the fibrils of some proteins [220], Thioflavin T, at the concentrations used in experiments, seems not to interfere with the fibril formation process significantly [221] because it binds to the specific sites that are not critical for secondary nucleation or elongation processes and does not block the whole fibril surface (so Equation (13) cannot be used for Thioflavin T). Other small molecules can also turn out to be inefficient inhibitors despite the strong binding.

Adsorption of inhibitor on aggregates will normally not reduce the fibril yield (theoretically, it even decreases the equilibrium monomer concentration). However, Chatani et al. [222] reported stabilization of the prefibrillar intermediate of the insulin B chain by fibrinogen due to binding to its surface. As a result, conversion into mature fibrils becomes thermodynamically blocked. In general, this is possible if the fibril formation mechanism is more complicated than the one we considered and includes the intermediate aggregates.

## 4. Conclusions

Design of novel fibril formation inhibitors can rely on one of three major molecular mechanisms of action, including binding with fibril-forming protein monomers, blocking the ends of the growing fibril, or binding to the surface of oligomers preventing nucleation.

Inhibitor-sequestering protein monomers can completely suppress fibril formation and disrupt the already-formed fibrils. For the structured proteins with well-defined binding sites, small-molecule ligands binding with high affinity and stabilizing the native state can be designed. This approach led to the development of the first-in-class clinically approved drug for the treatment of transthyretin amyloidosis. In the case of disordered proteins, strong binding with small molecules is less likely, but antibody-like proteins with high affinity can be designed.

The advantage of the inhibitors blocking fibril ends is their activity at low substoichiometric concentrations. Moreover, the design principles of peptides complementary to the self-recognition motif of pathogenic proteins, such as beta-amyloid, are well understood. However, these inhibitors do not prevent nucleation processes and lead to the accumulation of a large number of small aggregates that are feared to be very toxic.

Nucleation inhibitors are quite a novel idea with a limited number of proven examples and are not fully understood development principles. They can reduce the number of fibril particles favoring longer chains.
